# Comprehensive plasma metabolomics profiling develops diagnostic biomarkers of obstructive hypertrophic cardiomyopathy

**DOI:** 10.1186/s40364-025-00768-0

**Published:** 2025-04-05

**Authors:** Hao Cui, Yifan Wang, Xiumeng Hua, Jing Han, Han Mo, Shun Liu, Hongmei Wang, Siyuan Huang, Yiqi Zhao, Xiao Chen, Qian Zhao, Hao Jia, Yuan Chang, Jiangping Song

**Affiliations:** 1 State Key Laboratory of Cardiovascular Disease, Fuwai Hospital, National Center for Cardiovascular Diseases, Chinese Academy of Medical Sciences and Peking Union Medical College, Beijing, 100037 China; 2 Beijing Key Laboratory of Preclinical Research and Evaluation for Cardiovascular Implant Materials, Animal Experimental Centre, National Centre for Cardiovascular Disease, Fuwai Hospital, Chinese Academy of Medical Sciences and Peking Union Medical College, 167A Beilishi Road, Xi Cheng District, Beijing, 100037 China; 3 Department of Cardiac Surgery, Fuwai Hospital, National Center for Cardiovascular Diseases, Chinese Academy of Medical Sciences and Peking Union Medical College, Beijing, 100037 China; 4 Shenzhen Key Laboratory of Cardiovascular Disease, Fuwai Hospital Chinese Academy of Medical Sciences, Shenzhen, 518057 China; 5 Department of Cardiac Surgery, Fuwai Yunnan Hospital, Chinese Academy of Medical Sciences, Affiliated Cardiovascular Hospital of Kunming Medical University, Kunming, China

**Keywords:** Hypertrophic cardiomyopathy, Left ventricular hypertrophy, Biomarker, Metabolomics, Diagnosis

## Abstract

Hypertrophic cardiomyopathy (HCM) is the common cause of sudden cardiac death in young people and is characterized by cardiac hypertrophy. Non-HCM caused left ventricular hypertrophy (LVH) is more common in the population, especially in people with hypertension, obesity, and diabetes. In order to identify high-risk populations, a screening technique that can rapidly differentiate between HCM and LVH patients should be developed. Plasma metabolomics may help develop useful biomarkers for the disease diagnosis. We performed a comprehensive plasma metabolomic analysis on a total of 720 individuals, included 441 HCM patients, 160 LVH patients, and 119 normal controls (NC) (derivation cohort = 368, validation cohort = 352). Orthogonal partial least squares discriminant analysis (OPLS-DA) was used to construct discriminant models based on metabolomics, and the result showed significant changes in plasma metabolic profiling among the HCM, LVH, and NC. The prospective diagnostic biomarkers for HCM patients have been examined using variable importance in projection, fold change, and FDR. Acylcarnitines efficiently distinguished HCM and LVH patients, with a C14:0-carnitine AUC of 0.937 shown by the reiver operator characteristic (ROC) curve analysis. The biomarkers for the diagnosis of HCM patients was verified in another independent validation cohort. This study is the largest plasma metabolomics analysis of Chinese Han patients with HCM, finding biomarkers that can be used to distinguish between HCM from LVH patients. These results highlight the great potential value of plasma metabolic profiling analysis on HCM diagnoses.

**Supplementary information:**

The online version contains supplementary material available at 10.1186/s40364-025-00768-0.

## To the editor

Hypertrophic cardiomyopathy is characterized clinically by left ventricular hypertrophy and pathologically by cardiomyocyte enlargement and disarray, interstitial fibrosis, and inflammatory cell infiltration. (1–2) Patients with HCM may develop some complications and even sudden cardiac death, that may exert tremendous financial, social, and medical burdens on the healthcare system. (3–4) Screening of HCM depend on imaging modalities and genetic testing [5]. However, the expensive medical costs, time commitment, and reliance on expert supervision severely limit the widespread screening of HCM. Here, we perform metabolomic analysis with LC-MS on a total of 720 plasma samples. The derivation cohort was including 207 HCM patients, 104 LVH patients, and 57 NC, whereas validation cohort was including 234 HCM patients, 56 LVH patients, and 62 NC (Supplementary materials Tables 1,2; Supplementary materials Fig. 1A, B). A total of 406 metabolites were successfully identified (Supplementary materials Table 3).

**Fig. 1:**
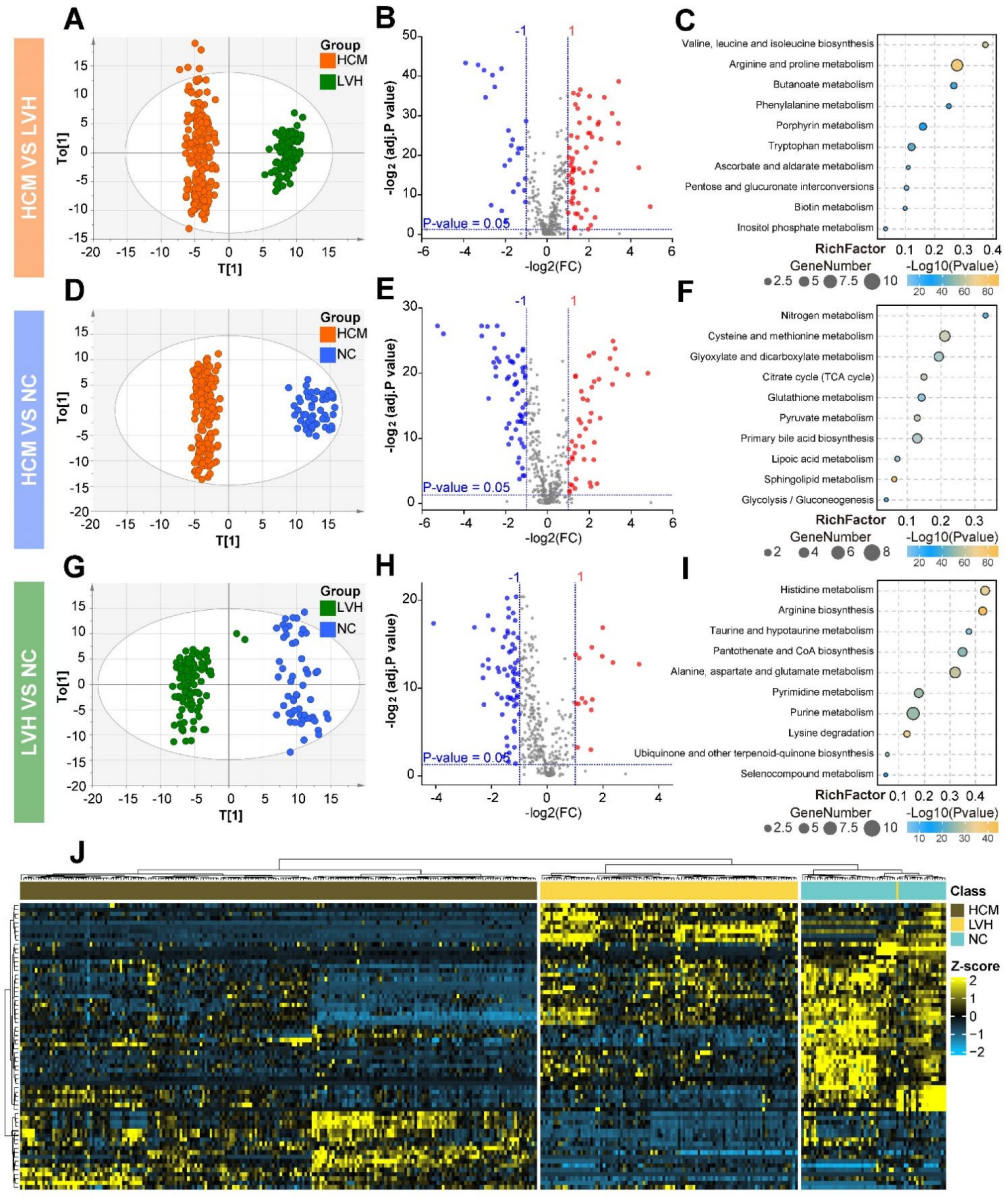
Plasma metabolic profiling of HCM patients, LVH patients, and NC. The OPLS-DA score plots of plasma metabolomes in HCM, LVH, and NC were shown as (**A**) HCM vs. LVH, (**B**) HCM vs. NC, and (**C**) LVH vs. NC in the derivation cohort. There were significant differences in the level of plasma metabolite between the two groups: (**D**) HCM vs. LVH, (**E**) HCM vs. NC, and (**F**) LVH vs. NC in the derivation cohort. Using Mann-Whitney U test, *P*-values adjusted by FDR, FDR < 0.05, are represented in the volcano plot, with red representing (fold change > 2) and blue representing (fold change < 0.5). KEGG pathways were enriched with significantly different metabolites between the two groups: (**G**) HCM vs. LVH, (**H**) HCM vs. NC, and (**I**) LVH vs. NC in the derivation cohort. All pathways are represented by bubbles. Each pathway was represented by bubbles. Bubble’s color and size were based on *P*-value and RichFactor. (**J**) A hierarchical clustering heatmap revealed significantly different expression metabolites across HCM, LVH, and NC in the derivation cohort. HCM, hypertrophic cardiomyopathy; LVH, left ventricular hypertrophy; NC, normal controls; OPLS-DA, orthogonal partial least squares discriminant analysis; FDR, false discovery rates; KEGG, kyoto encyclopedia of genes and genomes

The plasma metabolome revealed substantial differences across the HCM, LVH, and NC. An OPLS-DA revealed distinct metabolic differences between HCM vs. LVH (Fig. 1A), HCM vs. NC (Fig. 1B), and LVH vs. NC (Fig. 1C). All of the predicted variance, predictive ability, and CV-ANOVA *P* values suggested that the OPLS-DA model was robust and trustworthy (Supplementary materials Table 4). The permutation test demonstrated that the OPLS-DA models had not been overfitted (Supplementary materials Fig. 1C-E). We identified 83 differential metabolites in HCM vs. LVH, 103 in HCM vs. NC, and 79 in LVH vs. NC (Mann-Whitney U test, FDR < 0.05 and|FC| > 2) (Fig. 1D–F). The KEGG pathway analysis revealed that the differential metabolites in HCM vs. LVH, HCM vs. NC, and LVH vs. NC were enriched in arginine and proline metabolism, cysteine and methionine metabolism, and histidine metabolism, respectively (Fig. 1G-I). The result of unsupervised hierarchical clustering showed that the metabolic profiling of HCM was considerably different from the other two groups (Fig. 1J).

**Fig. 2:**
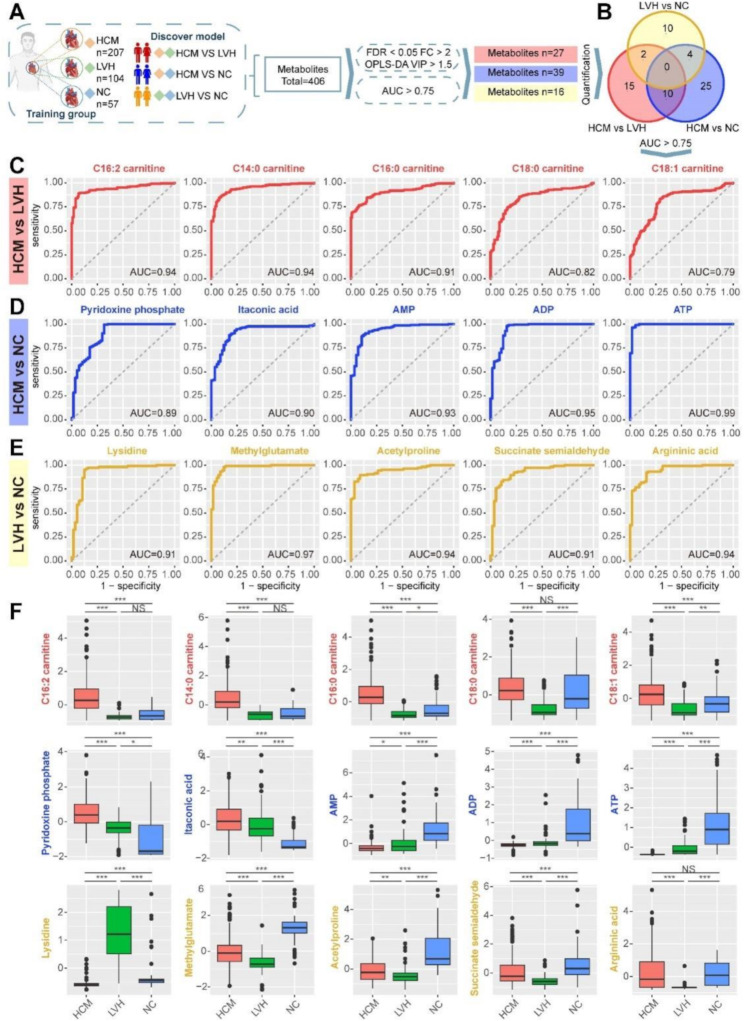
Prediction model based on plasma metabolome for HCM diagnosis in the derivation cohort. (**A**) Potential diagnostic biomarker screening procedure. (**B**) Venn diagram showing differential metabolites comparison between HCM, LVH, and NC groups in the derivation cohort. ROC curve analysis was performed to evaluate diagnostic effect between two groups in derivation cohort, (**C**) HCM vs. LVH, (**D**) HCM vs. NC, (**E**) LVH vs. NC in the derivation cohort. (**F**) Boxplot demonstrating the relative expression levels of the typical biomarkers for HCM, LVH, and NC in the derivation cohort. HCM, hypertrophic cardiomyopathy; LVH, left ventricular hypertrophy; NC, normal controls; ROC, receiver operator characteristic

The three groups were compared in pairs (HCM vs. LVH, HCM vs. NC, and LVH vs. NC) to screen the biomarkers through the several steps (Fig. 2A, B). The top five biomarkers which showed an excellently predictive effect with the AUC up to 0.94, 0.99, and 0.97 in the comparation of HCM vs. LVH, HCM vs. NC, and LVH vs. NC, respectively (Fig. 2C-E). Long-chain acylcarnitines demonstrated exceptionally strong and consistent diagnostic performance in distinguishing between HCM and LVH, and C14:0-carnitine exhibited the larger AUC (0.94; 95% CI, 0.91–0.96) than the others (Fig. 2C, F). We further investigated the gain effect of C14:0-carnitine on clinical parameters. The AUC of NT-proBNP alone was 0.71, while the AUC increased to 0.95 when combined with C14:0 carnitine (Supplementary materials Fig. 2). Compared with NC, plasma level of ATP, ADP, and AMP were significantly lower in HCM patients. This suggests that HCM is also characterized by abnormal energy metabolism, even if systolic dysfunction is not present. The metabolic differences between LVH and NC are predominantly focused on amino acid metabolism [6]. The metabolomic analysis was carried out independently in a validation cohort to confirm the predictive efficiency of the biomarkers. In the comparison of HCM vs. LVH, HCM vs. NC, and LVH vs. NC, the AUC values were 0.83–1.0, 0.89–0.99, and 0.6–1.0 (Supplementary materials Fig. 3). It was demonstrated that the application, reproducibility, and validity of predict performance of these biomarkers.

Metabolomics has been widely used in the research of cardiomyopathy, and it has the potential to provide considerable predictive value independent of traditional risk factors. (7–8) The biomarkers identified in this study, such as acylcarnitines, were also shown to be beneficial for DCM diagnosis [9]. Although of obvious importance, data on the application of metabolomics in HCM is scarce. The present study, with a larger sample size, used LVH patients as controls to fill this gap in metabolomics data in this field. In our previous studies, we have also successfully applied metabolomics to cardiomyopathies such as dilated cardiomyopathy, diabetic cardiomyopathy, and arrhythmogenic right ventricular cardiomyopathy [10–12]. These studies may provide important insights into the pathophysiology of cardiomyopathy. Nevertheless, our study has potential limitations, particularly with patients who were candidates for septal myectomy, it may not generalize to patients with mild clinical manifestations of HCM. Future use of prospective cohorts is required to validate the findings of this study.

## Electronic supplementary material

Below is the link to the electronic supplementary material.

Supplementary Material 1

## Data Availability

The data underlying this study are available from the corresponding author upon reasonable request.

## Abbreviations

HCM: Hypertrophic cardiomyopathy
LVH: Non-HCM caused left ventricular hypertrophy
NC: Normal control
LC-MS: Liquid chromatography-mass spectrometry
AUC: Area under curve
OPLS-DA: Orthogonal partial least squares discriminant analysis
FC: Fold change
FDR: False discovery rates
KEGG: Kyoto encyclopedia of genes and genomes
ATP: Adenosine triphosphate
ADP: Adenosine diphosphate
AMP: Adenosine monophosphate
DCM: Dilated cardiomyopathy
