# Supplementary material for: Comprehensive plasma metabolomics profiling develops diagnostic biomarkers of obstructive hypertrophic cardiomyopathy
Source: Biomark Res. 2025 Apr 5;13:55. doi: 10.1186/s40364-025-00768-0 (PMC11972456; doi:10.1186/s40364-025-00768-0)
Supplement: Supplementary file 1 — Supplementary Material 1 [file 40364_2025_768_MOESM1_ESM.docx]

**Supplementary Materials**

**Index**

**Expanded Methods**

**Supplementary Materials Figure 1.** **Metabolomic data assessment and descriptive analysis.**

**Supplementary Materials Figure 2.** **The diagnostic effects of the combination of NT-proBNP and C14:0 carnitine between HCM and LVH patients.**

**Supplementary Materials Figure 3.** **A validation cohort was used to evaluate the diagnostic effects of plasma biomarkers.**

**Supplementary Materials Table 1. Clinical characteristics of patients in the derivation and validation cohort.**

**Supplementary Materials Table 2. The program of gradient elution.**

**Supplementary Materials Table 3. The coefficients of variances of all identified metabolites.**

**Supplementary Materials Table 4. The reliability assessment of OPLS-DA model.**

**Expanded Methods**

**Chemicals and Reagents**

| Chemicals and Reagents | Source | Identifier |
| --- | --- | --- |
| L-carnitine-D_9_ | Cambridge Isotope Laboratories, Inc | CAS: 126827-79-0 |
| L-leucine-¹³C_6_ | Cambridge Isotope Laboratories, Inc | CAS: 201740-84-3 |
| D-Glucose-¹³C_6_ | Cambridge Isotope Laboratories, Inc | CAS: 201740-84-3 |
| Sodium D-3-hydroxybutyrate-¹³C_4_ | Cambridge Isotope Laboratories, Inc | CAS: 2483735-72-2 |
| Acetonitrile (ACN) | Merck Millipore | CAS: 75-05-8 |
| Methanol (MeOH) | Merck Millipore | CAS: 67-56-1 |

**Study Design and Sample Acquisition**

In this study, 720 individuals were collected from Fuwai Hospital, comprising 441 patients with obstructive HCM, 160 patients with non-HCM LVH, and 119 normal controls (NC). The diagnostic criteria of obstructive HCM were based on the 2024 ACC/AHA and 2023 ESC guidelines as follows: echocardiographic evidence of maximum LV wall thickness of ≥15 mm.^1-2^ Patients with previous septal myectomy and alcohol septal ablation were excluded. Clinical examination and echocardiography revealed no cardiac diseases in all normal controls. To minimize metabolite degradation, the fasting blood sample was processed within 1 hour to produce frozen plasma. Whole blood was centrifuged at 4°C for 10 minutes at 4000 rpm, and the plasma supernatant was collected and split into 0.2 mL Eppendorf tubes (70 μl plasma per tube). The plasma was immediately quenched in liquid nitrogen and stored at -80°C until analysis. This study was approved by the Ethics Committee of Fuwai Hospital and carried out in accordance with the Helsinki Declaration of 1964. The individuals who took part in this study provided written informed consent. The basic characteristics of individuals are provided in Supplementary materials Table 1.

**Metabolite Extraction from Plasma**

The extraction of plasma metabolites has previously been described.^3^ In summary, 50 μL of plasma was combined with 450 μL of extract liquor (H_2_O:ACN:MeOH, v/v/v, 2:4:4) and vortexed for 5 minutes. The mixture was then centrifuged at 18,400 g for 30 minutes at 4°C. The supernatant was collected, and then vacuum rotatory evaporator was used for the concentration. The particles were stored at -80°C and were dissolved in 100 μL of extract liquor until LC-MS analysis.

**LC-MS Analysis and Data Processing**

An untargeted metabolomic approach was performed using a Vanquish ultra-performance liquid chromatography system coupled to a Q-Exactive HF mass spectrometer (Thermo Fisher Scientific, Waltham, MA, USA). In brief, a gradient elution was applied for the separation of the metabolite. The ACQUITY BEH Amide column (150 × 2.1 mm, 1.7 μm, Waters, USA) were used for separation. The gradient elution and the composition of Solvent A and B were shown in Supplementary materials Table 2. The data was acquired in the fullMS-ddMS^2^ mode (the parent ion ranked in the top five). The following MS parameters were set: For the full scan mode, resolution 120,000, automatic gain control 2 × 10^6^, and maximum ion injection time 200 ms; for the MS/MS scan mode, resolution 15,000, automatic gain control 1 × 10^5^, maximum ion injection time 100 ms, dynamic exclusion 15s, and normalized collision energy 20, 40, 60 eV. Source ionization parameters were: spray voltage 3.5 kV for electrospray ionization positive (ESI+) and 4.0 kV for electrospray ionization negative (ESI-), capillary temperature set at 320°C, sheath gas set at 40, and aux gas set at 15.

The acquired raw instrument data (.raw) was processed using Compound Discoverer version 3.3 software (Thermo Fisher Scientific, Waltham, MA, USA). The process including deconvolution, peak alignment and extraction. The identification of metabolites using the Metabolomics Standards Initiative (MSI) defined four different levels of metabolite identification, including identified metabolites (level 1), putatively annotated compounds (level 2), putatively characterized compound classes (level 3), and unknown compounds (level 4). In this study, we applied level 1 and 2 annotations using the in-house standard database and the Human Metabolome Database, respectively. The in-house standard database was generated using ~600 commercial standards based on retention time, precursor ion, and MS/MS fragments. To adjust technical variables from metabolite extraction and LC-MS injection, four internal standards (L-carnitine-D_9_, L-leucine-¹³C6, D-Glucose-¹³C6, D-3-hydroxybutyrate-¹³C4) were spiked into the extraction solvent. The use of adding isotopic standards before and after metabolome extraction confirmed the reliability of our metabolomics approach, and the recovery rate of these internal standards were in the range of 80-120%. Equal volumes of each plasma sample were pooled and generated for quality control (QC) samples. To investigate intra-day and inter-day variation, QC samples added with the internal standards were collected as follows: for intra-day variation, in the morning (7:00–8:00), daytime (13:00–14:00), and evening (19:00–20:00); for inter-day variation, every two days for three times (days 1, 4, and 7). The variation is less than 5.0% for intra-day and 10% for inter-day assays that identified the stability of the LC-MS methods. Further, to detect any systematic errors during LC-MS analysis, QC samples were repetitively injected per ten samples. To calculate the coefficients of variation (CV) in QC samples, metabolites with a CV greater than 30 have been excluded from further analysis. Metabolite abundance was quantified using peak area and normalized using isotope-labeled standards (positive ion mode used L-carnitine-D_9_, negative ion mode used D-3-hydroxybutyrate-¹³C4).

**Statistical Analysis**

Orthogonal partial least squares discriminant analysis (OPLS-DA) was performed by SIMCA® version 15.0 software (Umetrics, Umeå, Sweden) with unit variance (UV) scaling before multivariate analysis. The R^2^Y and Q^2^ intercepts were used to evaluate the performance of OPLS-DA models. To prevent overfitting, a permutation test (n=200) was performed, and the consistency of fit was assessed using a cross-validated analysis of variance (CV-ANOVA). Metabolite set enrichment analysis (MSEA) was performed using MetaboAnalyst 5.0 (https://www.metaboanalyst.ca/), and the Kyoto Encyclopedia of Genes and Genomes database (KEGG) was used for the enrichment analysis. The unsupervised hierarchical clustering using Euclidean distance and ward.D2 linkage was employed and was constructed using the ComplexHeatmap (v2.6.2) package of R 4.0.5. The logistic regression (LR) algorithm was used to develop the predictive models based on NT-proBNP and C14:0 carnitine. The Receiver operating characteristic (ROC) analysis was performed using pROC (v.1.17.0) package in R version 4.0.5. The Kruskal-Wallis and Mann-Whitney U-tests were used to compare metabolite levels in three and two groups, respectively. The Benjamini–Hochberg method was used to correct for type I error and the false discovery rate (FDR) less than 0.05 were considered statistically significant. The categorical variables were compared using the chi-squared test, with p-values less than 0.05 indicating statistical significance. The significance test and LR were performed using SPSS 20.0 (IBM Corp., Armonk, NY, USA).

**Reference**

1. Ommen, S. R.; Ho, C. Y.; Asif, I. M.; Balaji, S.; Burke, M. A.; Day, S. M.; Dearani, J. A.; Epps, K. C.; Evanovich, L.; Ferrari, V. A.; Joglar, J. A.; Khan, S. S.; Kim, J. J.; Kittleson, M. M.; Krittanawong, C.; Martinez, M. W.; Mital, S.; Naidu, S. S.; Saberi, S.; Semsarian, C.; Times, S.; Waldman, C. B., 2024 AHA/ACC/AMSSM/HRS/PACES/SCMR Guideline for the Management of Hypertrophic Cardiomyopathy: A Report of the American Heart Association/American College of Cardiology Joint Committee on Clinical Practice Guidelines. *Circulation* **2024,** *149* (23), e1239-e1311.

2. Arbelo, E.; Protonotarios, A.; Gimeno, J. R.; Arbustini, E.; Barriales-Villa, R.; Basso, C.; Bezzina, C. R.; Biagini, E.; Blom, N. A.; de Boer, R. A.; De Winter, T.; Elliott, P. M.; Flather, M.; Garcia-Pavia, P.; Haugaa, K. H.; Ingles, J.; Jurcut, R. O.; Klaassen, S.; Limongelli, G.; Loeys, B.; Mogensen, J.; Olivotto, I.; Pantazis, A.; Sharma, S.; Van Tintelen, J. P.; Ware, J. S.; Kaski, J. P., 2023 ESC Guidelines for the management of cardiomyopathies. *European Heart Journal* **2023,** *44* (37), 3503-3626.

3. Cui, H.; Shu, S.; Li, Y.; Yan, X.; Chen, X.; Chen, Z.; Hu, Y.; Chang, Y.; Hu, Z.; Wang, X.; Song, J., Plasma Metabolites-Based Prediction in Cardiac Surgery-Associated Acute Kidney Injury. *J Am Heart Assoc* **2021,** *10* (22), e021825.

**Supplementary Materials Figure 1**


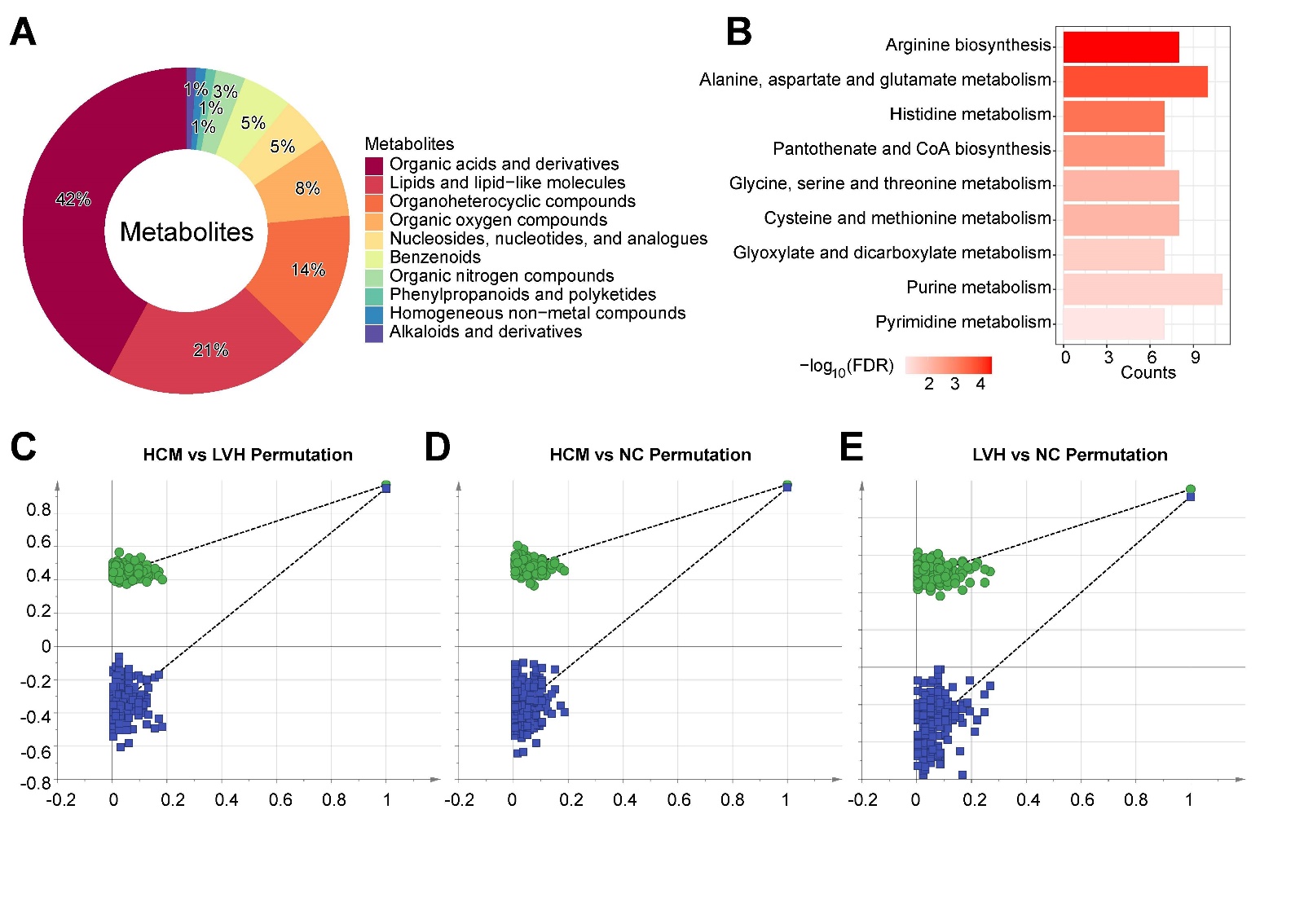
**Supplementary Materials Figure 1. Metabolomic data assessment and descriptive analysis.** **(A)** Pie chart of metabolite classification. Colors represent different compound classes, arranged counterclockwise in descending order of abundance. **(B)** KEGG enrichment analysis of metabolites, displaying the TOP 10 terms with the most significant FDR values. The permutation test (n=200) of OPLS-DA model between the two groups: **(C)** HCM vs LVH, **(D)** HCM vs NC, and **(E)** LVH vs NC. The x-axis represents the permutation retention rate of the permutation test, and the dots in the upper right corner represent the R² (green) and Q² (dark blue) values of the original model when the permutation retention rate is 1. R² measures the goodness of fit, while Q² measures the predictive ability of the model. Green dots represent the R² values obtained from the permutation test, while dark blue dots represent the Q² values obtained from the permutation test. The two dashed lines represent the regression lines of R² and Q², respectively. KEGG, kyoto encyclopedia of genes and genomes; OPLS-DA, orthogonal partial least squares discriminant analysis; HCM, hypertrophic cardiomyopathy; LVH, left ventricular hypertrophy; NC, normal controls.

**Supplementary Materials Figure 2**

**
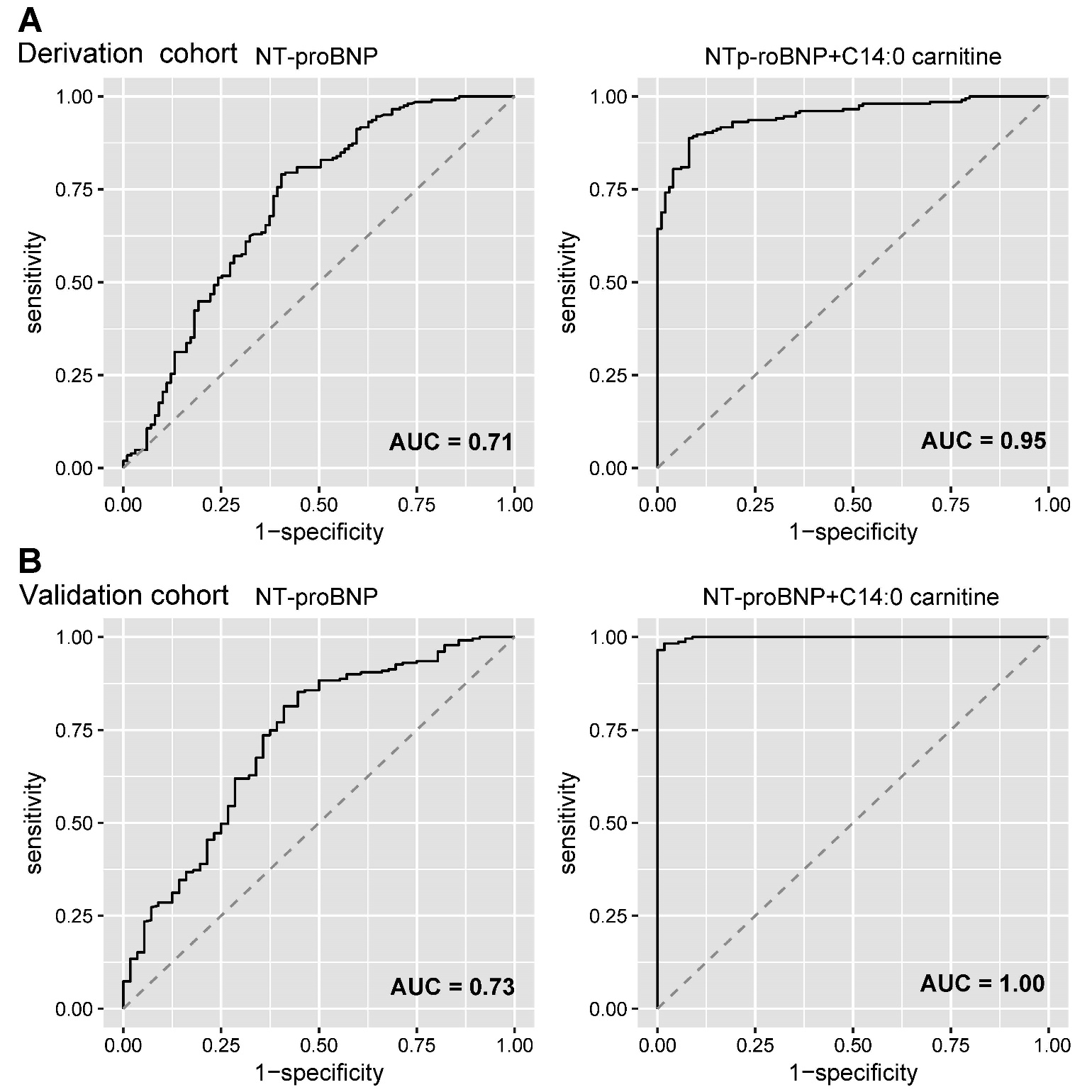
**

**Supplementary Materials Figure 2.** **The diagnostic effects of the combination of NT-proBNP and C14:0 carnitine between HCM and LVH patients.** The ROC curve was generated by NT-proBNP and NT-proBNP with C14:0 carnitine for differentiating HCM patients from LVH patients, (A) derivation cohort, (B) validation cohort. NT-proBNP, N-terminal pro-B-type natriuretic peptide; HCM, hypertrophic cardiomyopathy; LVH, left ventricular hypertrophy; ROC, receiver operator characteristic.

**Supplementary Materials Figure 3**

**
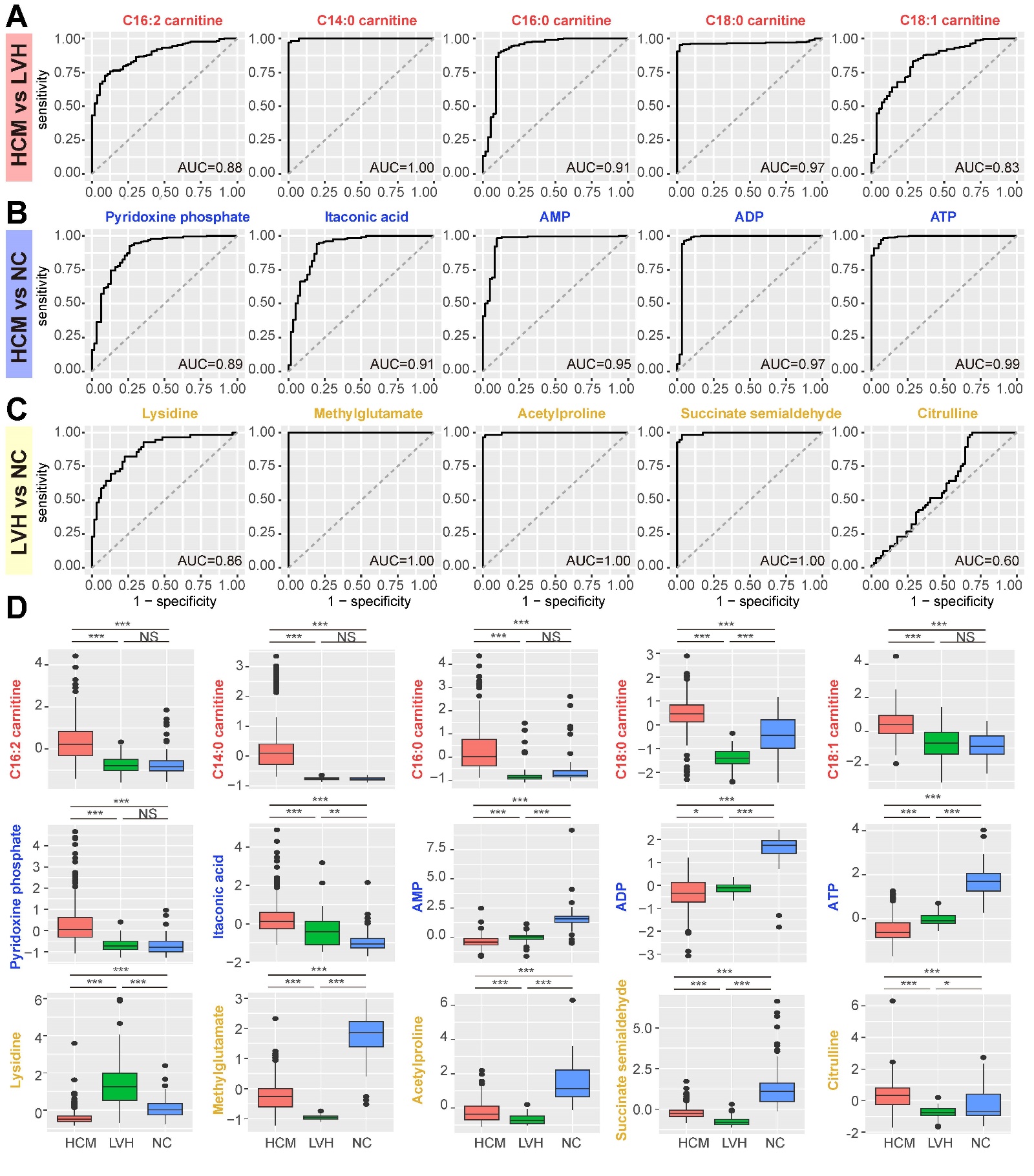
**

**Supplementary Materials Figure 3.** **A validation cohort was used to evaluate the diagnostic effects of plasma biomarkers.** ROC curve analysis was performed to evaluate diagnostic effect between two groups in validation cohort, **(A)** HCM vs. LVH, **(B)** HCM vs. NC, **(C)** LVH vs. NC. **(D)** Boxplots demonstrating the relative expression levels of the typical biomarkers for HCM, LVH, and NC in the validation cohort. ROC, receiver operator characteristic; HCM, hypertrophic cardiomyopathy; LVH, left ventricular hypertrophy; NC, normal controls.

**Supplementary Materials Table 1. Clinical characteristics of patients in the derivation and validation cohort.**

|  | **Derivation cohort** | | | **Validation cohort** | | |
| --- | --- | --- | --- | --- | --- | --- |
| **Name** | **HCM (n=207)** | **LVH (n=104)** | **P value** | **HCM (n=234)** | **LVH (n=56)** | **P value** |
| **Basic information** | |  |  |  |  |  |
| Male, n (%) | 119 (57) | 64 (62) | 0.493^#^ | 136 (58) | 27 (48) | 0.18^#^ |
| Age (years) | 51 (38–59) | 58 (49–65) | <0.001 | 51 (39–58) | 57 (47–65) | 0.008 |
| Body mass index, BMI (kg/m^2^) | 25 (23–28) | 26 (23–29) | 0.272 | 26 (23–28) | 24 (22–27) | 0.067 |
| Systolic blood pressure (mmHg) | 127 (113–139) | 130 (120–145) | 0.062 | 128 (116–140) | 132 (120–144) | 0.094 |
| Diastolic blood pressure (mmHg) | 72 (64–81) | 73 (68–80) | 0.456 | 74 (67–81) | 74 (65–80) | 0.423 |
| Heart rate (bpm) | 70 (62–80) | 78 (70–82) | <0.001 | 70 (64–78) | 76 (70–80) | 0.005 |
| Heart function classification, NYHA III-IV (%) | 69 (33) | 72 (31) | 0.564^#^ | 33 (32) | 17 (30) | 0.858^#^ |
| **Echocardiography** | |  |  |  |  |  |
| LVEF (%) | 70 (65–74) | 62 (55–65) | <0.001 | 66 (61–69) | 60 (57–65) | <0.001 |
| Ascending aorta diameter (mm) | 32 (29–35) | 34 (32–37) | <0.001 | 31 (28–34) | 34 (31–37) | <0.001 |
| LAD (mm) | 43 (40–48) | 43 (38–48) | 0.379 | 44 (40–49.5) | 43 (37–46) | 0.05 |
| LVEDD (mm) | 44 (40–48) | 56 (50–60) | <0.001 | 43 (40–46) | 55 (49–62) | <0.001 |
| IVS (mm) | 19 (17–23) | 10 (9–12) | <0.001 | 21 (18–26) | 10 (9–11) | <0.001 |
| LVPW (mm) | 11 (10–13) | 10 (9–11) | <0.001 | 12 (11–14) | 9 (8–11) | <0.001 |
| RVD (mm) | 23 (21–26) | 22 (20–24) | <0.001 | 22 (20–25) | 22 (21–25) | 0.984 |
| Main pulmonary diameter (mm) | 24 (22–26) | 24 (23–26) | 0.214 | 24 (22–26) | 25 (22–28) | 0.058 |
| Aortic valve systolic velocity (m/s) | 2.7 (1.7–4.3) | 1.5 (1.1–2.2) | <0.001 | 2.6 (1.9–4.3) | 1.4 (1.2–1.8) | <0.001 |
| Aortic valve systolic pressure difference (mmHG) | 29 (12–72) | 9 (4.8–19.8) | <0.001 | 36 (14–74) | 7.8 (5.8–12.3) | <0.001 |
| Pulmonary valve systolic velocity (m/s) | 1 (0.9–1.2) | 0.9 (0.8–1) | <0.001 | 1 (0.9–1.2) | 0.9 (0.8–1) | <0.001 |
| Pulmonary valve systolic pressure difference (mmHG) | 4 (3.2–5.8) | 3.2 (2.6–4) | <0.001 | 4 (3.2–5.8) | 3.2 (2.6–4) | <0.001 |
| LVOT gradient (mmHG) | 82 (67–98) |  |  | 81 (63–104) |  |  |
| **Blood parameters** | |  |  |  |  |  |
| Creatine (μmol/L) | 76 (67–87) | 83 (72–96) | <0.001 | 81 (69–90) | 81 (70–92) | 0.696 |
| Uric acid (μmol/L) | 395 (325–459) | 390 (334–452) | 0.9 | 381 (322–452) | 367 (293–444) | 0.253 |
| Glucose (mmol/L) | 5 (4.7–5.4) | 5.13 (4.62–6.43) | 0.266 | 4.88 (4.54–5.39) | 5.11 (4.4–6.40) | 0.226 |
| Triglycerides (mmol/L) | 1.09 (0.86–1.62) | 1.19 (0.95–1.83) | 0.086 | 1.2 (0.86–1.76) | 1.06 (0.86–1.45) | 0.343 |
| hs-CRP (mg/L) | 0.57 (0.35–1.25) | 1.31 (0.51–2.705) | <0.001 | 0.64 (0.34–1.50) | 0.83 (0.51–2.66) | 0.022 |
| Cholesterol (mmol/L) | 4.23 (3.58–5.04) | 3.74 (3.13–4.50) | <0.001 | 4.13 (3.49–4.68) | 4.09 (3.37–4.88) | 0.916 |
| HbA1c (%) | 5.55 (5.3–5.9) | 6 (5.4–6.7) | <0.001 | 5.4 (4.6–5.7) | 5.8 (5.5–6.7) | <0.001 |
| NT-proBNP (pg/ml) | 1150 (622–1999) | 453 (140–1121) | <0.001 | 1129 (566–2324) | 321 (120–1166) | <0.001 |

Continuous variables were presented as median (25th-75th percentile).

Continuous variables were used Mann Whitney U test.

# Categorical variables were used Chi-squared test.

NYHA, New York Heart Association; LVEF, Left ventricular ejection fraction; LVEDD, Left ventricular end diastolic diameter; IVS, Interventricular septum thickness; LVPW, Left ventricular posterior wall thickness; RVD, Right ventricular anteroposterior diameter; LAD, Left atrial anteroposterior diameter; LVOT, Left ventricular outflow tract; LVEDV, Left ventricular end diastolic volume; NT-proBNP, N-terminal pro-B-type natriuretic peptide; HbA1c, Glycosylated hemoglobin Type A1c; hs-CRP, High-sensitivity C-reactive protein CRP.

**Supplementary Materials Table 2. The program of gradient elution.**

| **Time (min)** | **Mobile phase A (%)** | **Mobile phase B (%)** |
| --- | --- | --- |
| 0 | 5 | 95 |
| 1.5 | 5 | 95 |
| 10.5 | 30 | 70 |
| 18 | 70 | 30 |
| 23 | 70 | 30 |
| 25 | 5 | 95 |
| 30 | 5 | 95 |

**Supplementary Materials Table 3. The coefficients of variances of all identified metabolites.**

| **Name** | **HMDB** | **Coefficients of Variances** | **Confidence Level** |
| --- | --- | --- | --- |
| Cholesteryl Acetate | HMDB0003822 | 5.2 | 1 |
| Decenoic Acid | HMDB0041012 | 13.6 | 1 |
| C16:0-Carnitine | HMDB0253127 | 18.1 | 1 |
| 3,4,5-Trimethoxycinnamic Acid | HMDB0002511 | 27.4 | 1 |
| 3-Hydroxybutyrylcarnitine | HMDB0013127 | 3.7 | 1 |
| PC(16:0/20:4) | HMDB0007983 | 10.8 | 1 |
| PC(16:0/18:2) | HMDB0007973 | 9.2 | 1 |
| PC(16:0/16:0) | HMDB0000564 | 3.3 | 1 |
| PC(18:1/16:0) | HMDB0008067 | 14.5 | 1 |
| 13(S)-Hotre | HMDB0244523 | 4.2 | 1 |
| 1-Aminocyclopropanecarboxylate | HMDB0036458 | 11.5 | 1 |
| 1-Methyladenosine | HMDB0003331 | 8.8 | 1 |
| 1-Methylguanine | HMDB0003282 | 3.6 | 1 |
| 1-Methylhistidine | HMDB0000001 | 6.2 | 1 |
| 2',3'-Cyclic Ump | HMDB0011640 | 13.5 | 1 |
| 2-Methylguanosine | HMDB0005862 | 8 | 1 |
| 3-Amino-4-Hydroxybenzoate | HMDB0304941 | 9.5 | 1 |
| 3-Methylcytosine | HMDB0011601 | 9.8 | 1 |
| 3-Methylglutarate | HMDB0000752 | 8.4 | 1 |
| 3-Methylhistamine | HMDB0001861 | 5.2 | 1 |
| 3-Methyl-Histidine | HMDB0000479 | 5 | 1 |
| 4-Guanidinobutanoate | HMDB0003464 | 4.6 | 1 |
| 4-Hydroxybenzaldehyde | HMDB0011718 | 3.4 | 1 |
| 4-Imidazoleacetate | HMDB0002024 | 12 | 1 |
| 5-Hydroxy-Tryptophan | HMDB0000472 | 5.5 | 1 |
| C16:2-Carnitine | HMDB0013334 | 12.3 | 1 |
| Acetylarginine | HMDB0004620 | 12.7 | 1 |
| Acetylhistamine | HMDB0013253 | 14.4 | 1 |
| Acetylmethionine | HMDB0011745 | 9.2 | 1 |
| Adenosine Diphosphate | HMDB0000061 | 4.2 | 1 |
| Adenosine Triphosphate | HMDB0000538 | 13 | 1 |
| Alanylthreonine | HMDB0028697 | 20.6 | 1 |
| Allothreonine | HMDB0004041 | 9.6 | 1 |
| Arginine | HMDB0000517 | 3 | 1 |
| Aspartate | HMDB0000191 | 3.9 | 1 |
| Biliverdin | HMDB0001008 | 19.5 | 1 |
| C4:1-Carnitine | HMDB0013126 | 6.3 | 1 |
| C12:0-Carnitine | HMDB0002250 | 8.5 | 1 |
| C14:0-Carnitine | HMDB0005066 | 5.5 | 1 |
| C18:0-Carnitine | HMDB0000848 | 5.7 | 1 |
| C8:0-Carnitine | HMDB0000791 | 13.1 | 1 |
| Caffeine | HMDB0001847 | 3.3 | 1 |
| Ceramide (d18:1/24:1(15Z)) | HMDB0004953 | 13.8 | 1 |
| C14:1-Carnitine | HMDB0002014 | 25.5 | 1 |
| Citrulline | HMDB0000904 | 5.7 | 1 |
| Creatine | HMDB0000064 | 23.5 | 1 |
| Creatinine | HMDB0000562 | 7.5 | 1 |
| Cystathionine | HMDB0000099 | 4.7 | 1 |
| Cystine | HMDB0000192 | 4.5 | 1 |
| Cytidine | HMDB0000089 | 7.4 | 1 |
| Cytosine | HMDB0000630 | 3.9 | 1 |
| C10:0-Carnitine | HMDB0000651 | 3.9 | 1 |
| Deoxycarnitine | HMDB0001161 | 3.8 | 1 |
| Deoxyuridine Monophosphate | HMDB0001409 | 12.6 | 1 |
| Diethanolamine | HMDB0004437 | 3.6 | 1 |
| Indole-3-Carboxylic Acid | HMDB0003320 | 8.2 | 1 |
| Argininic Acid | HMDB0003148 | 12.5 | 1 |
| Ornithine | HMDB0003374 | 6.3 | 1 |
| Glutarylcarnitine | HMDB0013130 | 9.1 | 1 |
| Glycine | HMDB0000123 | 7.8 | 1 |
| Glycochenodeoxycholate | HMDB0000637 | 8.6 | 1 |
| Glycocholate | HMDB0000138 | 7.9 | 1 |
| Guanidinoacetate | HMDB0000128 | 5.7 | 1 |
| Guanidinosuccinate | HMDB0003157 | 23.7 | 1 |
| Guanine | HMDB0000132 | 11.4 | 1 |
| Histamine | HMDB0000870 | 4 | 1 |
| Histidine | HMDB0000177 | 7.2 | 1 |
| Homocysteine | HMDB0000742 | 3.3 | 1 |
| Hypoxanthine | HMDB0000157 | 4.9 | 1 |
| Indole | HMDB0000738 | 13.9 | 1 |
| Indoline | HMDB0253472 | 5.9 | 1 |
| Inosine | HMDB0000195 | 7 | 1 |
| Kynurenine | HMDB0000684 | 5 | 1 |
| Alanine | HMDB0000161 | 19.2 | 1 |
| Cysteine | HMDB0000574 | 4.9 | 1 |
| Leucine | HMDB0000687 | 10.1 | 1 |
| Acetyl-Alanine | HMDB0000766 | 6.9 | 1 |
| Carnitine | HMDB0000062 | 11.7 | 1 |
| C6:0-Carnitine | HMDB0000756 | 5.2 | 1 |
| C18:1-Carnitine | HMDB0006469 | 5.6 | 1 |
| C16:1-Carnitine | HMDB0240774 | 6.2 | 1 |
| Lysidine |  | 7.4 | 1 |
| Lysine | HMDB0000182 | 4.2 | 1 |
| Methionine | HMDB0000696 | 7.9 | 1 |
| Methylguanidine | HMDB0001522 | 8.9 | 1 |
| Methylthioadenosine | HMDB0001173 | 5.6 | 1 |
| N,N-Dimethylarginine | HMDB0001539 | 4.6 | 1 |
| Acetylleucine | HMDB0011756 | 14.1 | 1 |
| Acetylmannosamine | HMDB0001129 | 11.3 | 1 |
| Acetyltryptophan | HMDB0013713 | 8.5 | 1 |
| Nicotinamide | HMDB0001406 | 14.2 | 1 |
| Methylglutamate | HMDB0062660 | 3.4 | 1 |
| Metylalanine | HMDB0094692 | 24.9 | 1 |
| Norleucine | HMDB0001645 | 3.3 | 1 |
| Norvaline | HMDB0013716 | 11.3 | 1 |
| C2-Carnitine | HMDB0000201 | 4 | 1 |
| Heptadecanoylcarnitine | HMDB0006210 | 3.5 | 1 |
| Heptanoylcarnitine | HMDB0013238 | 12.3 | 1 |
| Pantothenate | HMDB0000210 | 10 | 1 |
| Paraxanthine | HMDB0001860 | 15.2 | 1 |
| Phosphocreatine | HMDB0001511 | 3.9 | 1 |
| Phosphorylcholine | HMDB0001565 | 3.5 | 1 |
| Pipecolate | HMDB0000070 | 10.3 | 1 |
| Platelet-Activating Factor | HMDB0062195 | 6.5 | 1 |
| Proline | HMDB0000162 | 12.3 | 1 |
| C3-Carnitine | HMDB0000824 | 3.5 | 1 |
| Riboflavin | HMDB0000244 | 8.9 | 1 |
| Serotonin | HMDB0000259 | 7.2 | 1 |
| Sphinganine | HMDB0000269 | 9.6 | 1 |
| Taurine | HMDB0000251 | 12.9 | 1 |
| 4-Hydroxy-Proline | HMDB0000725 | 12 | 1 |
| Trigonelline | HMDB0000875 | 3.8 | 1 |
| Tryptophan | HMDB0000929 | 13.9 | 1 |
| Tyrosine | HMDB0000158 | 10.2 | 1 |
| Uracil | HMDB0000300 | 9.9 | 1 |
| Urobilin | HMDB0004160 | 10.9 | 1 |
| Valine | HMDB0000883 | 22.6 | 1 |
| Glycolaldehyde Dimer | HMDB0003344 | 17.7 | 1 |
| 2-Keto-3-Deoxy-D-Gluconic Acid | HMDB0001353 | 8.3 | 1 |
| Kynurenate | HMDB0000715 | 7.6 | 1 |
| Aconitate | HMDB0000958 | 5.8 | 1 |
| 2-Methylmaleate | HMDB0000634 | 24.5 | 1 |
| Tagatose | HMDB0003418 | 3.1 | 1 |
| Aminoadipate | HMDB0000510 | 13.4 | 1 |
| Pyroglutamate | HMDB0000267 | 8.5 | 1 |
| Ethylmalonate | HMDB0000622 | 13.4 | 1 |
| Mevalolactone | HMDB0006024 | 11.6 | 1 |
| Serine | HMDB0000187 | 6.7 | 1 |
| Citramalate | HMDB0000426 | 3.4 | 1 |
| Methyl Acetoacetate | HMDB0000310 | 18.8 | 1 |
| 5-Hydroxyindoleacetate | HMDB0000763 | 12.8 | 1 |
| Acetyllysine | HMDB0000446 | 8.9 | 1 |
| Raffinose | HMDB0003213 | 3.8 | 1 |
| 3-Hydroxybutyrate | HMDB0000011 | 3.2 | 1 |
| 3,4-Dihydroxyphenylacetate | HMDB0001336 | 25.3 | 1 |
| Acetylglycine | HMDB0000532 | 7.9 | 1 |
| Xylose | HMDB0000098 | 10.2 | 1 |
| Dihydrouracil | HMDB0000076 | 19.9 | 1 |
| N-Acetylphenylalanine | HMDB0000512 | 12.5 | 1 |
| Glutamine | HMDB0000641 | 7.9 | 1 |
| Indole-3-Acetate | HMDB0000197 | 4 | 1 |
| Urate | HMDB0000289 | 2.7 | 1 |
| Glyceraldehyde | HMDB0001051 | 6.8 | 1 |
| Acetylproline | HMDB0094701 | 2.9 | 1 |
| Xanthosine | HMDB0000299 | 8.1 | 1 |
| Quinaldic Acid | HMDB0000842 | 12.1 | 1 |
| Sebacate | HMDB0000792 | 3.1 | 1 |
| N-Acetylserine | HMDB0002931 | 13.3 | 1 |
| Adenosine Monophosphate | HMDB0000045 | 4.8 | 1 |
| Homovanillate | HMDB0000118 | 3.2 | 1 |
| Glutamate | HMDB0000148 | 4.2 | 1 |
| Salicylate | HMDB0001895 | 12.4 | 1 |
| Urocanate | HMDB0000301 | 9.4 | 1 |
| Threonine | HMDB0000167 | 7.6 | 1 |
| Citrate | HMDB0000094 | 7.7 | 1 |
| Succinate Semialdehyde | HMDB0001259 | 6.7 | 1 |
| Hippurate | HMDB0000714 | 25.8 | 1 |
| Xanthurenate | HMDB0000881 | 15.1 | 1 |
| 4-Pyridoxate | HMDB0000017 | 20 | 1 |
| Phenylalanine | HMDB0000159 | 13.5 | 1 |
| Asparagine | HMDB0000168 | 10.2 | 1 |
| Hydroxyphenyllactic Acid | HMDB0000755 | 27.2 | 1 |
| Glucose | HMDB0000122 | 8.4 | 1 |
| Pentanoate | HMDB0000892 | 20.8 | 1 |
| 12(13)-Dhome | HMDB0004705 | 8.1 | 1 |
| 9-Hpode | HMDB0062434 | 15.6 | 1 |
| Malic Acid | HMDB0000156 | 6.2 | 1 |
| N-Acetylthreonine | HMDB0062557 | 4.1 | 1 |
| Lactate | HMDB0000190 | 9.9 | 1 |
| Muricholic Acid | HMDB0000865 | 22.8 | 1 |
| 12(13)Ep-9-Kode | HMDB0013623 | 3 | 1 |
| 3-Hydroxyoctanoic Acid | HMDB0001954 | 21.7 | 1 |
| N-Lactoyl-Glycine | HMDB0062183 | 27.6 | 1 |
| Allantoic Acid | HMDB0001209 | 11.4 | 1 |
| Methyl-Lactate | HMDB0254599 | 3.7 | 1 |
| Cholic Acid | HMDB0000619 | 7.7 | 1 |
| 2-Keto-Glutaramic Acid | HMDB0001552 | 3.5 | 1 |
| Glyceric Acid | HMDB0006372 | 26.1 | 1 |
| Methylcysteine | HMDB0002108 | 3.6 | 1 |
| Fumaric Acid | HMDB0000134 | 9.9 | 1 |
| Gamma-Aminobutyric Acid | HMDB0000112 | 13.2 | 1 |
| Glutaric Acid | HMDB0000661 | 9.3 | 1 |
| Hypotaurine | HMDB0000965 | 11.4 | 1 |
| Itaconic Acid | HMDB0002092 | 3.5 | 1 |
| Ergothioneine | HMDB0003045 | 7.2 | 1 |
| Succinamic Acid | HMDB0258538 | 12.5 | 1 |
| 4-Oxo-Proline | HMDB0246561 | 15.8 | 1 |
| Maleamic Acid | HMDB0254310 | 18.5 | 1 |
| Myristoleic Acid | HMDB0002000 | 3.2 | 1 |
| N-Methylhistidine | HMDB0255176 | 10.1 | 1 |
| Acetylornithine | HMDB0003357 | 11.9 | 1 |
| Methyllysine | HMDB0002038 | 6.5 | 1 |
| Acetylhistidine | HMDB0032055 | 3.2 | 1 |
| Phosphohydroxypyruvic Acid | HMDB0001024 | 11.5 | 1 |
| Methyl-Proline | HMDB0094696 | 7.4 | 1 |
| Pyridoxine Phosphate | HMDB0001319 | 4.5 | 1 |
| Pyroglutamine | HMDB0062558 | 16.8 | 1 |
| Pyruvic Acid | HMDB0000243 | 11.3 | 1 |
| S-Allylcysteine | HMDB0034323 | 13.6 | 1 |
| Succinic Anhydride | HMDB0032523 | 11.7 | 1 |
| Thymine | HMDB0000262 | 7 | 1 |
| Glutaconic Acid | HMDB0000620 | 6.1 | 1 |
| Uridine | HMDB0000296 | 5.3 | 1 |
| Valylvaline | HMDB0029140 | 13.8 | 1 |
| Vitamin C | HMDB0000044 | 5 | 1 |
| PC(18:0/22:6) | HMDB0008727 | 4.2 | 2 |
| PC(20:4/20:4) | HMDB0008477 | 6.6 | 2 |
| PC(16:1/16:1) | HMDB0008002 | 6.7 | 2 |
| 1,3-Dicyclohexylurea | HMDB0244166 | 13 | 2 |
| 1,6-Hexanediamine | HMDB0244244 | 4.1 | 2 |
| PC(18:1/22:6) | HMDB0008123 | 16 | 2 |
| PC(18:1/14:0) | HMDB0008097 | 5.7 | 2 |
| 1-Methylinosine | HMDB0002721 | 3.6 | 2 |
| PC(18:0/20:3) | HMDB0008046 | 11.8 | 2 |
| PC(18:1/18:2) | HMDB0256161 | 4.1 | 2 |
| PC(16:0/18:3) | HMDB0008166 | 11.1 | 2 |
| PC(O-18:0/20:4) | HMDB0013420 | 4.8 | 2 |
| PC(14:0/20:4) | HMDB0007884 | 6.6 | 2 |
| 2-Hexenoylcarnitine | HMDB0013161 | 5.2 | 2 |
| 2-Hydroxyhippuric Acid | HMDB0000840 | 5.9 | 2 |
| C14:2-Carnitine | HMDB0013331 | 6 | 2 |
| 3,4-Diaminopyridine | HMDB0246022 | 4 | 2 |
| 3-Hydroxytetradecadiencarnitine | HMDB0013332 | 3 | 2 |
| 3-Hydroxytetradecenoylcarnitine | HMDB0013330 | 5.3 | 2 |
| 3-Hydroxydodecanoylcarnitine | HMDB0061638 | 6.3 | 2 |
| 3-Hydroxyhexadecadienoylcarnitine | HMDB0013335 | 5 | 2 |
| 3-Hydroxylidocaine | HMDB0060655 | 9.5 | 2 |
| 3-Hydroxyoctanoylcarnitine | HMDB0241694 | 7.8 | 2 |
| 3-Hydroxytetradecanoylcarnitine | HMDB0061640 | 5.9 | 2 |
| 3-Methylenenorleucine |  | 18.6 | 2 |
| 3-Methylglutarylcarnitine | HMDB0000552 | 10.4 | 2 |
| 4-Acetamidoantipyrin | HMDB0246326 | 10.6 | 2 |
| 4-Hydroxyprolyllysine | HMDB0028868 | 4.4 | 2 |
| C10:1-Carnitine | HMDB0013205 | 11.9 | 2 |
| Acacetin Diacetate |  | 4.6 | 2 |
| Acpc | HMDB0015135 | 8.8 | 2 |
| Adonixanthin |  | 5.1 | 2 |
| Ala-Pro | HMDB0028695 | 23 | 2 |
| Aminoproline | HMDB0030405 | 5.9 | 2 |
| Anacardic Acid | HMDB0033896 | 10.9 | 2 |
| Asp-Lys | HMDB0004985 | 11.6 | 2 |
| Asp-Ser | HMDB0028762 | 5.6 | 2 |
| Ala-Lys | HMDB0028692 | 6.8 | 2 |
| C20 Sphingomyelin (d18:1/20:0) | HMDB0012102 | 7.8 | 2 |
| Capryloylglycine | HMDB0000832 | 3.5 | 2 |
| Cgmp | HMDB0001314 | 7.1 | 2 |
| Cyclohexylamine | HMDB0031404 | 5.4 | 2 |
| Morphine | HMDB0014440 | 9 | 2 |
| Cycloserine | HMDB0014405 | 10.4 | 2 |
| Desethylamiodarone | HMDB0060528 | 6.9 | 2 |
| Desvenlafaxine | HMDB0015646 | 9.6 | 2 |
| Dexamethasone | HMDB0015364 | 9.7 | 2 |
| DG(16:1/18:3) | HMDB0007273 | 8.2 | 2 |
| Diethyl Acetamidomalonate |  | 3.8 | 2 |
| Dihydrocodeine | HMDB0250396 | 11.1 | 2 |
| Dihydrothymine | HMDB0000079 | 12.8 | 2 |
| Dihydrouridine | HMDB0000497 | 5.6 | 2 |
| 5-Aminoindole | HMDB0000214 | 6.4 | 2 |
| Dihydroxy-2-Naphthoic Acid | HMDB0013609 | 3.4 | 2 |
| Glu-Gly | HMDB0028819 | 5.6 | 2 |
| Glu-Ser | HMDB0028828 | 9.8 | 2 |
| Glu-Thr | HMDB0028829 | 5 | 2 |
| Gly-Asp | HMDB0028837 | 7.6 | 2 |
| Gly-Phe | HMDB0028848 | 10 | 2 |
| Gly-Pro | HMDB0000721 | 17 | 2 |
| Gly-Lys | HMDB0028846 | 11.9 | 2 |
| Lecithin | HMDB0002159 | 9.3 | 2 |
| Leucoline | HMDB0033731 | 10.6 | 2 |
| Leu-Pro | HMDB0011175 | 26.4 | 2 |
| Lidocaine | HMDB0014426 | 4.2 | 2 |
| Lys-Gly | HMDB0028951 | 10.3 | 2 |
| Lys-Leu | HMDB0028955 | 3.4 | 2 |
| Lysolecithin | HMDB0010384 | 3.3 | 2 |
| LysoPC(0:0/18:0) | HMDB0011128 | 5.7 | 2 |
| LysoPC(18:3) | HMDB0010387 | 10 | 2 |
| LysoPC(20:5) | HMDB0010397 | 7.5 | 2 |
| LysoPC(22:1) | HMDB0010399 | 15.4 | 2 |
| LysoPC(22:4) | HMDB0010401 | 7.9 | 2 |
| LysoPC(22:5) | HMDB0010402 | 8.3 | 2 |
| LysoPC(P-18:0) | HMDB0013122 | 3.4 | 2 |
| LysoPC(14:1) | HMDB0010380 | 4.4 | 2 |
| Gly-Leu | HMDB0000759 | 4.3 | 2 |
| Acetylspermidine | HMDB0002189 | 4 | 2 |
| 2-Pentanamido-3-Phenylpropanoic Acid | HMDB0094646 | 12.2 | 2 |
| Ribosylhistidine | HMDB0002089 | 4.7 | 2 |
| PC(18:3/18:2) | HMDB0008204 | 4.9 | 2 |
| PC(20:5/P-18:1) | HMDB0008523 | 11.7 | 2 |
| PC(O-18:0/18:2) | HMDB0013418 | 8.2 | 2 |
| PC(O-18:1/18:2) | HMDB0013429 | 3.5 | 2 |
| PC(O-20:1/20:4) | HMDB0013444 | 22.2 | 2 |
| PC(P-16:0/16:0) | HMDB0011206 | 3.8 | 2 |
| PC(P-16:0/18:3) | HMDB0008225 | 17.4 | 2 |
| Propanolamine |  | 8.7 | 2 |
| SM(d18:0/22:3) | HMDB0013468 | 9 | 2 |
| Tetranor 12-Hete | HMDB0060055 | 4 | 2 |
| Tilarginine | HMDB0029416 | 6.7 | 2 |
| Urobilinogen | HMDB0004158 | 16.6 | 2 |
| Castanospermine | HMDB0249700 | 13.7 | 2 |
| 12-Hydroxylauric Acid | HMDB0002059 | 11.3 | 2 |
| 2-Ethylhexyl Maleate |  | 9.3 | 2 |
| 2-Hydroxyisovaleric Acid | HMDB0000407 | 9.9 | 2 |
| 2-Pyrrolidone | HMDB0002039 | 3 | 2 |
| 3-Acetamidopropanal | HMDB0012880 | 6.2 | 2 |
| 3-Butyl-4-Hydroxyanisole | HMDB0059925 | 11.5 | 2 |
| 3-Hydroxysebacic Acid | HMDB0000350 | 7 | 2 |
| 3-Hydroxytetradecanedioic Acid | HMDB0000394 | 3.9 | 2 |
| 3-Oxotetradecanoic Acid | HMDB0010730 | 9.8 | 2 |
| 4-Methyleneglutamic Acid | HMDB0029433 | 3.1 | 2 |
| 5-Hydroxyisouric Acid | HMDB0030097 | 2.9 | 2 |
| 6-Deoxocastasterone | HMDB0033984 | 5.4 | 2 |
| 8-Chlorotheophyline | HMDB0247429 | 13.2 | 2 |
| 8-Hydroxy-7-Methylguanine | HMDB0006037 | 11.2 | 2 |
| 8-Hydroxyquercetagetin | HMDB0030159 | 6.1 | 2 |
| Acamprosate | HMDB0014797 | 10.2 | 2 |
| Acefylline | HMDB0247895 | 6.5 | 2 |
| Aceglutamide | HMDB0006029 | 8.7 | 2 |
| Acetanilide | HMDB0001250 | 2.9 | 2 |
| Acrylic Acid | HMDB0031647 | 20.4 | 2 |
| Ala-Ser | HMDB0028696 | 28 | 2 |
| Albendazole Sulfone | HMDB0060561 | 8.7 | 2 |
| Allicin | HMDB0033963 | 12.3 | 2 |
| Aspartyl-4-Hydroxyproline | HMDB0011160 | 6 | 2 |
| Hydroxytriazolam | HMDB0061053 | 13.7 | 2 |
| Aminocaproic Acid | HMDB0001901 | 17.2 | 2 |
| Aspartyl-Proline | HMDB0002335 | 7.1 | 2 |
| Asp-Gln | HMDB0028751 | 6.1 | 2 |
| Aspirin | HMDB0001879 | 4.4 | 2 |
| Asulam | HMDB0248680 | 6.3 | 2 |
| Aspartylaspartic Acid | HMDB0028749 | 8.7 | 2 |
| Ethyl Glucuronide | HMDB0010325 | 20.4 | 2 |
| Galactopyranose | HMDB0037209 | 6.9 | 2 |
| Bicine | HMDB0011727 | 16.3 | 2 |
| Bilirubin | HMDB0000054 | 6.6 | 2 |
| Butanoic Anhydride | HMDB0303914 | 17.9 | 2 |
| Captopril-Cysteine | HMDB0060562 | 4.1 | 2 |
| Cholic Acid Glucuronide | HMDB0002577 | 2.9 | 2 |
| Cidofovir | HMDB0014513 | 3.2 | 2 |
| Cinnamic Acid | HMDB0000567 | 3 | 2 |
| Crotonic Acid | HMDB0010720 | 9.6 | 2 |
| Cyclohexanecarboxylic Acid | HMDB0031342 | 3.7 | 2 |
| Alanyl-Alanine | HMDB0003459 | 9.3 | 2 |
| Deoxycholic Acid | HMDB0000626 | 7.2 | 2 |
| DG(14:1/16:1) | HMDB0007041 | 8.7 | 2 |
| D-Glucopyranuronic Acid | HMDB0000127 | 10.2 | 2 |
| Diethylpyrocarbonate | HMDB0032873 | 19.8 | 2 |
| Dioctyl Hexanedioate | HMDB0041619 | 13.8 | 2 |
| Dihydrolipoic Acid | HMDB0012210 | 11.8 | 2 |
| Diisodecyl Phthalate | HMDB0251351 | 5.6 | 2 |
| Diisooctyl Sebacate |  | 4.8 | 2 |
| Dimethyl Fumarate | HMDB0031257 | 10.3 | 2 |
| Diphenol Glucuronide | HMDB0059998 | 27.3 | 2 |
| Dimethyllysine | HMDB0341183 | 6.8 | 2 |
| 3-Mercaptopicolinic Acid | HMDB0245916 | 4.5 | 2 |
| Oxalylglycine | HMDB0255221 | 5.8 | 2 |
| Threo-3-Phenylserine | HMDB0002184 | 7.7 | 2 |
| Ethylenediaminetetraacetic Acid | HMDB0015109 | 12.6 | 2 |
| Ethylparaben | HMDB0032573 | 9.1 | 2 |
| Furfural | HMDB0032914 | 8.5 | 2 |
| Gamma-Glu-His | HMDB0029151 | 3.6 | 2 |
| Glucoheptonic Acid | HMDB0252766 | 10.3 | 2 |
| Glutaral | HMDB0029599 | 22.6 | 2 |
| Aspartate-Semialdehyde | HMDB0012249 | 6.4 | 2 |
| Succinoylpyridine | HMDB0000992 | 2.9 | 2 |
| His-Asp | HMDB0028881 | 23.3 | 2 |
| 2-Amino-4-Propyl-5-Phosphono-3-Pentenoate | HMDB0243638 | 12.9 | 2 |
| Hostmaniane | HMDB0032796 | 8.4 | 2 |
| Hydroxyhexanoate | HMDB0001624 | 19.1 | 2 |
| Hydroxynorleucine |  | 5.4 | 2 |
| Indoleacetyl Glutamic Acid | HMDB0038665 | 3.7 | 2 |
| Ethylcysteine | HMDB0001890 | 12.1 | 2 |
| Methylbutanethioic Acid | HMDB0061879 | 3.8 | 2 |
| Iminodiacetic Acid | HMDB0011753 | 29.9 | 2 |
| Erythrulose | HMDB0006293 | 3.3 | 2 |
| Lauryl Aldehyde | HMDB0033933 | 10.2 | 2 |
| Cysteinylglycine Disulfide | HMDB0000709 | 7.8 | 2 |
| Leu-Gln | HMDB0028927 | 6.5 | 2 |
| Leu-Val | HMDB0028942 | 6.3 | 2 |
| Levonorgestrel | HMDB0014511 | 7.6 | 2 |
| Levosimendan | HMDB0015058 | 3.4 | 2 |
| LysoPE(22:6) | HMDB0011526 | 7.3 | 2 |
| Lys-Tyr | HMDB0028963 | 4.5 | 2 |
| Madecassic Acid | HMDB0036670 | 24.1 | 2 |
| Hydroxyenterolactone | HMDB0041650 | 3 | 2 |
| Carboxymethyllysine | HMDB0240347 | 6.3 | 2 |
| Acetyl-Zonisamide | HMDB0060603 | 17.3 | 2 |
| Nitrosomorpholine | HMDB0255208 | 24.2 | 2 |
| 3-Aminoisobutanoic Acid | HMDB0003911 | 3.9 | 2 |
| Propylurea | HMDB0255240 | 8.8 | 2 |
| Stearoyl-Aspartate | HMDB0241939 | 6.3 | 2 |
| Inositol 1,3,4,5-Tetrakisphosphate | HMDB0001059 | 4.8 | 2 |
| Phenylglyoxylic Acid | HMDB0001587 | 10.9 | 2 |
| Phosphorous Acid | HMDB0001443 | 3.4 | 2 |
| Ser-Glu | HMDB0029038 | 3.7 | 2 |
| Sulfuric Acid | HMDB0001448 | 8 | 2 |
| Tetrahydrofuran | HMDB0000246 | 26.7 | 2 |
| Thr-Pro | HMDB0029069 | 4.6 | 2 |
| Traumatic Acid | HMDB0000933 | 3.6 | 2 |
| Trihydroxycoprostanic Acid | HMDB0000601 | 5.2 | 2 |
| Trihydroxycoprostanoic Acid | HMDB0002163 | 5.6 | 2 |
| Trp-Phe | HMDB0029090 | 5.2 | 2 |
| Tyrosyltyrosine | HMDB0029117 | 11 | 2 |
| Val-Glu | HMDB0029126 | 11.3 | 2 |
| Val-Ser | HMDB0029136 | 13.3 | 2 |
| Vanillyl Mandelic Acid | HMDB0259761 | 23.9 | 2 |
| Varanic Acid | HMDB0002195 | 9.9 | 2 |
| Vigabatrin | HMDB0015212 | 20.2 | 2 |

**Supplementary Materials Table 4. The reliability assessment of OPLS-DA model.**

| **OPLS-DA model** | **R^2^Y** | **Q^2^** | **CV-ANOVA P value** |
| --- | --- | --- | --- |
| HCM vs. LVH | 0.972 | 0.952 | < 0.001 |
| HCM vs. NC | 0.974 | 0.954 | < 0.001 |
| LVH vs. NC | 0.951 | 0.914 | < 0.001 |

HCM, Hypertrophic cardiomyopathy; LVH, Non-HCM caused left ventricular hypertrophy; NC, Normal control
